# Whole-genome sequences of *Campylobacter coli* and *Campylobacter jejuni* isolates from rhesus macaques (*Macaca mulatta*) with and without intestinal disease

**DOI:** 10.1128/mra.00018-24

**Published:** 2024-03-06

**Authors:** Rebecca L. Bacon, Keri N. Norman, Colette A. Nickodem, Javier A. Vinasco, Stanton B. Gray, Carolyn L. Hodo, Sara D. Lawhon

**Affiliations:** 1 Department of Veterinary Pathobiology, School of Veterinary Medicine & Biomedical Sciences, Texas A&M University, College Station, Texas, USA; 2 Department of Veterinary Integrative Biosciences, School of Veterinary Medicine & Biomedical Sciences, Texas A&M University, College Station, Texas, USA; 3 Michale E. Keeling Center for Comparative Medicine and Research, The University of Texas MD Anderson Cancer Center, Bastrop, Texas, USA; Rochester Institute of Technology, Rochester, New York, USA

**Keywords:** *Campylobacter jejuni*, *Campylobacter coli*, rhesus macaque, intestinal disease, post-infectious irritable bowel, chronic enterocolitis

## Abstract

*Campylobacter jejuni* or *Campylobacter coli* infection can lead to post-infectious irritable bowel syndrome in humans and may produce a similar syndrome in rhesus macaques (*Macaca mulatta*). We report the complete genomes of 8 *C*. *jejuni* isolates and 103 *C*. *coli* isolates obtained from rhesus macaques with and without intestinal disease.

## ANNOUNCEMENT

*Campylobacter jejuni* and *Campylobacter coli* are leading causes of bacterial gastroenteritis, and following infection, ∼20% of people will develop post-infectious irritable bowel syndrome (PI-IBS) (1–4). Rhesus macaques (*Macaca mulatta*) (RM) can also experience acute campylobacteriosis, and there is evidence that they develop a PI-IBS-like syndrome resulting in chronic diarrhea (5). There are gene-level differences between *Campylobacter* strains that produce PI-IBS and those that do not, but sparse reports of whole-genome sequences (WGS) from *Campylobacter* isolates obtained from RM (6–9). We present 8 WGS of *C. jejuni* and 103 WGS of *C. coli* from RM with and without intestinal disease.

Swabs of RM feces were collected during wellness exams, hospitalization, or necropsy at the Michale E. Keeling Center for Comparative Medicine and Research. Animals were cared for in accordance with the USDA Animal Welfare Act and established IACUC policies. Swabs were used to inoculate Campy blood agar selective media (Remel, ThermoFisher Scientific, Waltham, MA, USA) which was incubated at 41°C–43°C in a microaerophilic environment for 48–72 hours. The microaerophilic environment was generated using a GasPak EZ Gas Generating Pouch System (BD, Franklin Lakes, NJ, USA). Colonies suspected of being *Campylobacter* species were Gram-stained and tested with a commercially available oxidase reagent consisting of a 1% aqueous solution of N,N,N′,N′-tetramethyl-p-phenylenediamine dihydrochloride (BD). Characteristic Gram-negative, oxidase-positive colonies were sub-cultured and frozen at −80°C in *Brucella* broth (BD) with 10% glycerol, transported to the Texas A&M University (TAMU) Clinical Microbiology Laboratory where they were revived on blood agar and *Campylobacter* selective media plates consisting of Campy-Cefex plates (BD) and modified charcoal cefoperazone deoxycholate agar, made from blood free *Campylobacter* selectivity agar (HiMedia, Mumbai, India), supplemented with 8 mg/L cefoperazone, 4 mg/L teicoplanin, and 10 mg/L amphotericin B (HiMedia), and incubated at 42°C for 48 hours in a microaerophilic environment as described above. Isolates were speciated using matrix-assisted, laser desorption and ionization, time-of-flight (MALDI-TOF) mass spectrometry according to the manufacturer’s protocol (Biotyper, Bruker Daltonics Inc., Billerica, MA, USA) using FlexFirmware v.3.4.206.94 with Compass software v.4.1.100.10 and BDAL library v.11897. After additional sub-culturing on blood agar plates to ensure purity, DNA was extracted using the QIAcube HT DNA extraction platform and QIAamp 96 DNA QIAcube HT kit following the manufacturer’s instructions. DNA quality and quantity were evaluated using 2 µL of DNA on a LVis plate with a FLUOstar Omega multiplate reader. All samples fell within an acceptable 260/280 nm ratio range and were stored at −20°C.

Double-stranded (ds) DNA concentrations were assessed using a Qubit dsDNA high-sensitivity assay kit and Qubit 4 fluorometer. Libraries were prepared with IDT xGen DNA library prep kit and UDI Normalase primers (IDT, Coralville, IA, USA) following the manufacturer’s protocol. Library quality was assessed using the Qubit 4 fluorometer and NGS standard sensitivity fragment analysis kit (Agilent Technologies, Santa Clara, CA, USA) with a Fragment Analyzer automated capillary electrophoresis system. The pooled libraries were loaded at 13.5 pM concentration into an Illumina MiSeq V2 500-cycle cartridge and sequenced on the Illumina MiSeq platform using 2 × 250 paired-end reads (10, 11). Bioinformatics were completed using the TAMU High-Performance Research Computing system. All bioinformatics utilized the software default parameters. Quality of reads and a summary information report were obtained using FastQC v.0.11.9 and MultiQC v.1.7 (12, 13). Indexes and adapters were trimmed with Trimmomatic v.0.39, sequences assembled with St. Petersburg genome assembler (SPAdes) v.3.14.0, and quality assessed with Quality Assessment Tool (QUAST) (14–16). Sequences were included in further analysis if the genome size was 1.6 Mb, coverage depth was at least 20×, and guanine-cytosine content (GC)-content was at least 30.4%. Multilocus sequence typing (MLST) was performed using MLST v.2.1.17, and antimicrobial resistance genes, point mutations, plasmids, and virulence factors were detected using Abricate v.0.9.9 (17–20).

Assemblies ranged from 12 to 708 contigs with a total length between 1,604,706 bp and 1,839,536 bp, and *N*_50_ contig lengths of 3,246–322,944 with an average GC-content of 31.2%. A list of assemblies and GenBank accession numbers is in given Table 1.

**TABLE 1:** Characteristics and accession numbers of genome sequences of *Campylobacter jejuni* and *C. coli* isolates from rhesus macaques with and without intestinal disease

Sample	Organism	Host intestinal disease	No. of contigs	*N*_50_ (bp)	Coverage (%)	Genome size (bp)	G + C content (%)	GenBank accession no.
2	*C. jejuni*	Yes	20	178,959	36.33324	1,676,018	30.46	SAMN38909928
10	*C. jejuni*	No	37	175,354	46.84625	1,787,441	30.18	SAMN38909936
52	*C. jejuni*	No	14	322,944	47.28748	1,690,736	30.44	SAMN38909977
56	*C. jejuni*	No	37	198,078	34.58232	1,788,868	30.18	SAMN38909981
69	*C. jejuni*	No	32	198,078	30.19519	1,782,502	30.19	SAMN38909994
74	*C. jejuni*	No	153	20,821	31.58797	1,801,038	30.18	SAMN38909999
104	*C. jejuni*	Yes	425	5,796	23.45778	1,651,546	30.54	SAMN38910028
108	*C. jejuni*	No	203	15,826	28.62104	1,803,331	30.19	SAMN38910032
1	*C. coli*	No	27	171,129	31.53036	1,690,046	31.34	SAMN38909927
3	*C. coli*	No	34	174,774	35.09052	1,736,591	31.3	SAMN38909929
4	*C. coli*	No	32	126,735	41.7259	1,760,530	31.17	SAMN38909930
5	*C. coli*	No	34	174,774	51.48681	1,736,183	31.3	SAMN38909931
6	*C. coli*	No	39	137,763	37.23116	1,735,726	31.3	SAMN38909932
7	*C. coli*	No	22	138,429	39.28135	1,678,825	31.35	SAMN38909933
8	*C. coli*	No	29	223,285	38.24534	1,719,861	31.32	SAMN38909934
9	*C. coli*	Yes	16	163,417	42.63004	1,640,223	31.33	SAMN38909935
11	*C. coli*	No	17	162,890	45.64465	1,641,468	31.33	SAMN38909937
12	*C. coli*	No	28	162,934	50.17786	1,756,281	31.25	SAMN38909938
13	*C. coli*	No	55	219,887	49.73176	1,724,146	31.27	SAMN38909939
14	*C. coli*	No	26	162,722	41.24713	1,755,300	31.25	SAMN38909940
15	*C. coli*	No	76	46,661	27.49452	1,759,776	31.25	SAMN38909941
16	*C. coli*	No	49	162,722	40.41303	1,764,989	31.25	SAMN38909942
17	*C. coli*	No	21	171,840	36.79243	1,760,458	31.17	SAMN38909943
18	*C. coli*	No	24	171,840	36.56247	1,760,220	31.17	SAMN38909944
19	*C. coli*	No	24	163,139	49.10859	1,759,470	31.17	SAMN38909945
20	*C. coli*	No	21	170,094	32.29894	1,712,054	31.29	SAMN38909946
21	*C. coli*	No	24	162,784	45.51724	1,805,429	31.17	SAMN38909947
22	*C. coli*	No	25	213,134	44.59417	1,694,405	31.38	SAMN38909948
23	*C. coli*	No	24	213,171	34.83324	1,694,410	31.38	SAMN38909949
24	*C. coli*	No	23	171,840	34.06028	1,760,882	31.17	SAMN38909950
25	*C. coli*	No	25	163,139	49.15716	1,760,503	31.17	SAMN38909951
26	*C. coli*	No	23	219,887	56.13047	1,713,011	31.29	SAMN38909952
27	*C. coli*	No	23	163,139	49.34708	1,759,641	31.18	SAMN38909953
28	*C. coli*	No	26	166,124	35.8322	1,759,383	31.17	SAMN38909954
29	*C. coli*	No	23	166,125	36.9341	1,760,552	31.17	SAMN38909955
30	*C. coli*	No	25	172,962	34.30232	1,736,186	31.29	SAMN38909956
31	*C. coli*	No	31	170,158	48.23171	1,717,170	31.28	SAMN38909957
32	*C. coli*	No	26	171,840	38.11838	1,760,376	31.17	SAMN38909958
33	*C. coli*	No	28	162,722	42.68903	1,755,383	31.25	SAMN38909959
34	*C. coli*	No	22	213,130	49.47311	1,693,437	31.38	SAMN38909960
35	*C. coli*	Yes	54	141,878	38.90146	1,835,925	31.05	SAMN38909961
36	*C. coli*	No	53	163,138	46.69221	1,838,855	31.05	SAMN38909962
37	*C. coli*	No	21	173,085	41.75475	1,694,178	31.38	SAMN38909963
38	*C. coli*	No	18	162,899	46.71169	1,640,479	31.33	SAMN38909964
39	*C. coli*	No	26	191,425	36.16686	1,694,004	31.39	SAMN38909965
40	*C. coli*	No	50	163,138	33.63427	1,836,712	31.05	SAMN38909966
41	*C. coli*	No	114	26,993	31.51573	1,775,071	31.26	SAMN38909967
43	*C. coli*	Yes	30	130,459	38.74353	1,756,246	31.25	SAMN38909968
44	*C. coli*	No	23	213,094	40.14966	1,687,016	31.35	SAMN38909969
45	*C. coli*	No	27	171,182	50.29948	1,689,967	31.33	SAMN38909970
46	*C. coli*	Yes	19	213,978	35.64178	1,687,528	31.35	SAMN38909971
47	*C. coli*	No	197	16,049	32.64219	1,753,234	31.3	SAMN38909972
48	*C. coli*	No	25	171,358	49.87025	1,690,398	31.33	SAMN38909973
49	*C. coli*	No	24	172,761	47.48012	1,760,204	31.17	SAMN38909974
50	*C. coli*	No	23	213,131	48.91516	1,695,954	31.38	SAMN38909975
51	*C. coli*	Yes	36	173,869	46.29216	1,787,510	31.1	SAMN38909976
53	*C. coli*	No	21	227,075	42.37155	1,724,318	31.28	SAMN38909978
54	*C. coli*	No	21	162,784	43.14547	1,783,574	31.19	SAMN38909979
55	*C. coli*	No	26	171,129	50.09584	1,690,363	31.33	SAMN38909980
57	*C. coli*	No	29	163,203	37.41251	1,715,841	31.28	SAMN38909982
58	*C. coli*	No	26	171,502	54.94049	1,694,743	31.34	SAMN38909983
59	*C. coli*	No	31	277,722	45.17455	1,733,614	31.3	SAMN38909984
60	*C. coli*	No	99	30,067	28.27295	1,746,453	31.29	SAMN38909985
61	*C. coli*	No	69	55,048	32.84235	1,763,171	31.18	SAMN38909986
62	*C. coli*	No	117	27,649	39.18951	1,697,081	31.35	SAMN38909987
63	*C. coli*	No	15	224,657	51.92761	1,686,797	31.32	SAMN38909988
64	*C. coli*	No	27	162,722	34.57401	1,755,616	31.25	SAMN38909989
65	*C. coli*	No	36	130,246	35.09756	1,688,658	31.35	SAMN38909990
66	*C. coli*	No	33	277,721	49.50106	1,735,728	31.3	SAMN38909991
67	*C. coli*	No	23	220,546	52.62372	1,712,850	31.28	SAMN38909992
68	*C. coli*	No	20	213,130	44.56276	1,694,130	31.38	SAMN38909993
70	*C. coli*	No	26	129,808	33.6299	1,759,343	31.18	SAMN38909995
71	*C. coli*	No	48	163,138	46.49842	1,839,536	31.05	SAMN38909996
72	*C. coli*	Yes	176	17,664	35.40454	1,801,899	31.1	SAMN38909997
73	*C. coli*	No	27	172,735	32.43503	1,733,279	31.3	SAMN38909998
75	*C. coli*	No	21	171,840	35.76447	1,759,998	31.17	SAMN38910000
76	*C. coli*	No	20	174,868	38.15458	1,733,266	31.3	SAMN38910001
77	*C. coli*	No	20	171,840	45.9947	1,760,501	31.17	SAMN38910002
78	*C. coli*	No	20	171,840	53.67061	1,759,523	31.17	SAMN38910003
79	*C. coli*	No	27	162,722	41.70427	1,756,310	31.25	SAMN38910004
80	*C. coli*	No	26	162,722	47.5371	1,756,769	31.25	SAMN38910005
81	*C. coli*	No	24	163,139	39.1238	1,759,730	31.17	SAMN38910006
82	*C. coli*	No	26	171,412	30.8332	1,689,861	31.33	SAMN38910007
83	*C. coli*	No	26	170,157	34.59428	1,715,657	31.28	SAMN38910008
84	*C. coli*	Yes	20	213,130	41.0762	1,694,031	31.38	SAMN38910009
85	*C. coli*	No	12	169,030	40.13134	1,640,383	31.33	SAMN38910010
86	*C. coli*	No	22	220,546	36.63291	1,674,130	31.34	SAMN38910011
87	*C. coli*	Yes	24	224,309	36.81201	1,720,059	31.32	SAMN38910012
88	*C. coli*	Yes	34	173,872	38.91469	1,788,615	31.1	SAMN38910013
89	*C. coli*	No	32	130,214	41.59637	1,688,924	31.35	SAMN38910014
90	*C. coli*	No	25	213,131	41.21321	1,694,036	31.38	SAMN38910015
91	*C. coli*	No	26	171,129	47.72139	1,689,664	31.33	SAMN38910016
92	*C. coli*	No	24	171,183	44.0521	1,690,180	31.33	SAMN38910017
93	*C. coli*	No	16	163,417	59.37339	1,640,735	31.33	SAMN38910018
94	*C. coli*	No	22	213,132	46.5128	1,693,804	31.39	SAMN38910019
95	*C. coli*	Yes	62	47,796	38.17798	1,665,532	31.35	SAMN38910020
96	*C. coli*	Yes	15	168,803	41.71994	1,641,970	31.33	SAMN38910021
97	*C. coli*	No	17	132,442	39.55955	1,640,762	31.33	SAMN38910022
98	*C. coli*	No	126	21,077	31.34463	1,653,977	31.33	SAMN38910023
99	*C. coli*	No	77	44,603	26.26655	1,759,371	31.25	SAMN38910024
101	*C. coli*	Yes	299	8,571	23.19147	1,688,153	31.38	SAMN38910025
102	*C. coli*	Yes	708	3,246	19.73854	1,604,706	31.85	SAMN38910026
103	*C. coli*	No	357	8,204	28.62789	1,767,427	31.3	SAMN38910027
105	*C. coli*	Yes	50	118,206	28.55247	1,732,767	31.3	SAMN38910029
106	*C. coli*	Yes	197	17,178	22.6367	1,784,405	31.12	SAMN38910030
107	*C. coli*	Yes	46	110,034	26.98624	1,731,639	31.3	SAMN38910031
109	*C. coli*	Yes	28	165,011	33.32153	1,688,229	31.35	SAMN38910033
110	*C. coli*	No	45	96,842	23.48991	1,755,927	31.25	SAMN38910034
111	*C. coli*	Yes	96	32,675	31.80805	1,697,583	31.34	SAMN38910035
112	*C. coli*	No	27	162,722	37.08843	1,755,705	31.25	SAMN38910036
113	*C. coli*	Yes	29	162,722	35.59599	1,756,445	31.25	SAMN38910037

## Data Availability

This project has been deposited in NCBI under the BioProject accession number PRJNA1054170 at https://www.ncbi.nlm.nih.gov/bioproject/1054170 and includes GenBank accession numbers SAMN38909927 through SAMN38910037.

## References

[1] Costa D, Iraola G. 2019. Pathogenomics of emerging Campylobacter species. Clin Microbiol Rev 32:e00072-18. doi:10.1128/CMR.00072-18 31270126 PMC6750134

[2] Goddard MR, O’Brien S, Williams N, Guitian J, Grant A, Cody A, Colles F, Buffet J-C, Adlen E, Stephens A, Godfray HCJ, Maiden MCJ. 2022. A restatement of the natural science evidence base regarding the source, spread and control of Campylobacter species causing human disease. Proc Biol Sci 289:20220400. doi:10.1098/rspb.2022.0400 35703046 PMC9198779

[3] Spiller R, Garsed K. 2009. Postinfectious irritable bowel syndrome. Gastroenterology 136:1979–1988. doi:10.1053/j.gastro.2009.02.074 19457422

[4] Riddle MS, Gutierrez RL, Verdu EF, Porter CK. 2012. The chronic gastrointestinal consequences associated with Campylobacter. Curr Gastroenterol Rep 14:395–405. doi:10.1007/s11894-012-0278-0 22864805

[5] Laing ST, Merriam D, Shock BC, Mills S, Spinner A, Reader R, Hartigan-O’Connor DJ. 2018. Idiopathic colitis in rhesus macaques is associated with dysbiosis, abundant enterochromaffin cells and altered T-cell cytokine expression. Vet Pathol 55:741–752. doi:10.1177/0300985818780449 29929446

[6] Peters S, Pascoe B, Wu Z, Bayliss SC, Zeng X, Edwinson A, Veerabadhran-Gurunathan S, Jawahir S, Calland JK, Mourkas E, Patel R, Wiens T, Decuir M, Boxrud D, Smith K, Parker CT, Farrugia G, Zhang Q, Sheppard SK, Grover M. 2021. Campylobacter jejuni genotypes are associated with post-infection irritable bowel syndrome in humans. Commun Biol 4:1015. doi:10.1038/s42003-021-02554-8 34462533 PMC8405632

[7] Kim SG, Summage-West CV, Sims LM, Foley SL. 2021. Complete genome sequence of Campylobacter fetus subsp. venerealis P4531 from a rhesus monkey. Microbiol Resour Announc 10:e0073921. doi:10.1128/MRA.00739-21 34672709 PMC8530035

[8] Kim SG, Summage-West CV, Sims LM, Wu L, Kim J, Nho S, Foley SL. 2022. Complete genome sequence of Campylobacter coli strain P4581, a hybrid carrying Campylobacter jejuni genomic content. Microbiol Resour Announc 11:e0084722. doi:10.1128/mra.00847-22 36047780 PMC9583788

[9] Weis AM, Storey DB, Taff CC, Townsend AK, Huang BC, Kong NT, Clothier KA, Spinner A, Byrne BA, Weimer BC. 2016. Genomic comparison of Campylobacter spp. and their potential for zoonotic transmission between birds, primates, and livestock. Appl Environ Microbiol 82:7165–7175. doi:10.1128/AEM.01746-16 27736787 PMC5118927

[10] Ceric O, Tyson GH, Goodman LB, Mitchell PK, Zhang Y, Prarat M, Cui J, Peak L, Scaria J, Antony L, et al.. 2019. Enhancing the one health initiative by using whole genome sequencing to monitor antimicrobial resistance of animal pathogens: vet-LIRN collaborative project with veterinary diagnostic laboratories in United States and Canada. BMC Vet Res 15:130. doi:10.1186/s12917-019-1864-2 31060608 PMC6501310

[11] Tyson GH, Ceric O, Guag J, Nemser S, Borenstein S, Slavic D, Lippert S, McDowell R, Krishnamurthy A, Korosec S, et al.. 2021. Genomics accurately predicts antimicrobial resistance in Staphylococcus pseudintermedius collected as part of vet-LIRN resistance monitoring. Vet Microbiol 254:109006. doi:10.1016/j.vetmic.2021.109006 33581494 PMC11555765

[12] Andrews S. 2010. FastQC: a quality control tool for high throughput sequence data. Available from: http://www bioinformatics babraham ac uk/projects/fastqc

[13] Ewels P, Magnusson M, Lundin S, Käller M. 2016. MultiQC: summarize analysis results for multiple tools and samples in a single report. Bioinformatics 32:3047–3048. doi:10.1093/bioinformatics/btw354 27312411 PMC5039924

[14] Bolger AM, Lohse M, Usadel B. 2014. Trimmomatic: a flexible trimmer for illumina sequence data. Bioinformatics 30:2114–2120. doi:10.1093/bioinformatics/btu170 24695404 PMC4103590

[15] Prjibelski A, Antipov D, Meleshko D, Lapidus A, Korobeynikov A. 2020. Using spades de novo assembler. Curr Protoc Bioinformatics 70:e102. doi:10.1002/cpbi.102 32559359

[16] Gurevich A, Saveliev V, Vyahhi N, Tesler G. 2013. QUAST: quality assessment tool for genome assemblies. Bioinformatics 29:1072–1075. doi:10.1093/bioinformatics/btt086 23422339 PMC3624806

[17] Florensa AF, Kaas RS, Clausen P, Aytan-Aktug D, Aarestrup FM. 2022. ResFinder–an open online resource for identification of antimicrobial resistance genes in next-generation sequencing data and prediction of phenotypes from genotypes. Microb Genom 8:000748. doi:10.1099/mgen.0.000748 35072601 PMC8914360

[18] Chen L, Zheng D, Liu B, Yang J, Jin Q. 2016. VFDB 2016: hierarchical and refined dataset for big data analysis—10 years on. Nucleic Acids Res 44:D694–D697. doi:10.1093/nar/gkv1239 26578559 PMC4702877

[19] Carattoli A, Zankari E, García-Fernández A, Voldby Larsen M, Lund O, Villa L, Møller Aarestrup F, Hasman H. 2014. In silico detection and typing of plasmids using plasmidfinder and plasmid multilocus sequence typing. Antimicrob Agents Chemother 58:3895–3903. doi:10.1128/AAC.02412-14 24777092 PMC4068535

[20] Zankari E, Allesøe R, Joensen KG, Cavaco LM, Lund O, Aarestrup FM. 2017. PointFinder: a novel web tool for WGS-based detection of antimicrobial resistance associated with chromosomal point mutations in bacterial pathogens. J Antimicrob Chemother 72:2764–2768. doi:10.1093/jac/dkx217 29091202 PMC5890747

